# Multidisciplinary approach in children with autism spectrum disorder

**DOI:** 10.1192/j.eurpsy.2021.256

**Published:** 2021-08-13

**Authors:** A.C. Stanciu, F. Rad, I. Mihailescu, L. Mateescu, R. Grozavescu, E. Andrei, B. Budisteanu, F. Linca, D. Ioana, C. Iliescu, S. Papuc, A. Arghir, I. Dobrescu, M. Budisteanu

**Affiliations:** 1 Faculty Of Medicine, ‘Carol Davila’ University of Medicine and Pharmacy, Bucharest, Romania; 2 Department Of Child Psychiatry, ‘Carol Davila’ University of Medicine and Pharmacy; ‘Prof. Dr. Alexandru Obregia’ Clinical Hospital of Psychiatry, Bucharest, Romania; 3 Department Of Child Psychiatry, ‘Prof. Dr. Alexandru Obregia’ Clinical Hospital of Psychiatry, Bucharest, Romania; 4 Genetics Laboratory, ‘Victor Babes’ National Institute of Pathology, Bucharest, Romania; 5 Department Of Child Psychiatry, ‘Victor Babes’ National Institute of Pathology; ‘Prof. Dr. Alexandru Obregia’ Clinical Hospital of Psychiatry; ‘Titu Maiorescu’ University, Faculty of Medicine, Bucharest, Romania

**Keywords:** autism spectrum disorder, intellectual disability, multidisciplinary, comorbidities

## Abstract

**Introduction:**

Autism spectrum disorder (ASD) is characterized by persistent deficits in social communication and social interaction across multiple contexts and it is marked by repetitive sensory–motor behaviours and restricted interests or activities. Now recognized to occur in up to 1% of the population, the prevalence of ASD has registered a steady increase in the past two decades. Heterogeneity of presentation is a hallmark with comorbid psychiatric and medical morbidities frequently reported. Comorbidities mask and delay the diagnosis and are the cause of inadequate therapies.

**Objectives:**

In the present paper, we studied a cohort of patients with ASD, investigating the rates and types of psychiatric and medical comorbidities.

**Methods:**

A retrospective study of psychiatric and medical comorbidities was carried out on a sample of 120 participants that met ASD criteria according to DSM-V. The patients were examined with a detailed medical history, physical examination, as well as some additional functional, imaging, laboratory and genetic investigations. The associated conditions considered were: attention deficit/hyperactivity disorder (ADHD), epilepsy, intellectual disability, gastrointestinal symptoms, ophtalmologic manifestations, infections.

**Results:**

Of the 120 ASD subjects referred, 25 (20.8%) received the diagnosis of epilepsy. ADHD was established in 24 cases (20%). IQ score was obtained in half of the patients, 43.3% of them presenting a severe intellectual disability (IQ<35). Respiratory disorders occured in 25% of the cases. Ophtalmological findings were observed in 9.1% of the cases. Other frequent comorbidities included motor disturbances and feeding problems.

**Conclusions:**

A better understanding of comorbidities in ASD patients improves interdisciplinary collaboration, thus facilitating effective treatment programs.

**Disclosure:**

No significant relationships.

